# Sequential stream segregation in normally-hearing and cochlear-implant listenersa)

**DOI:** 10.1121/1.4973516

**Published:** 2017-01-04

**Authors:** Viral D. Tejani, Kara C. Schvartz-Leyzac, Monita Chatterjee

**Affiliations:** Department of Hearing and Speech Sciences, University of Maryland, Cochlear Implants and Psychophysics Laboratory, 0100 LeFrak Hall, College Park, Maryland 20742, USA

## Abstract

Sequential stream segregation by normal hearing (NH) and cochlear implant (CI) listeners was investigated using an irregular rhythm detection (IRD) task. Pure tones and narrowband noises of different bandwidths were presented monaurally to older and younger NH listeners via headphones. For CI users, stimuli were delivered as pure tones via soundfield and via direct electrical stimulation. Results confirmed that tonal pitch is not essential for stream segregation by NH listeners and that aging does not reduce NH listeners' stream segregation. CI listeners' stream segregation was significantly poorer than NH listeners' with pure tone stimuli. With direct stimulation, however, CI listeners showed significantly stronger stream segregation, with a mean normalized pattern similar to NH listeners, implying that the CI speech processors possibly degraded acoustic cues. CI listeners' performance on an electrode discrimination task indicated that cues that are salient enough to make two electrodes highly discriminable may not be sufficiently salient for stream segregation, and that gap detection/discrimination, which must depend on perceptual electrode differences, did not play a role in the IRD task. Although the IRD task does not encompass all aspects of full stream segregation, these results suggest that some CI listeners may demonstrate aspects of stream segregation.

## I. INTRODUCTION

Sounds comprising a complex listening environment are perceptually attributed to their respective sources via the process termed “auditory scene analysis” (Bregman, 1990). Acoustic cues used by the auditory system to parse the auditory scene include source-differences in pitch, harmonicity, timbre, and onset times. A common concern of patients with hearing impairment (HI) is continued difficulty understanding speech in background noise even when the overall signal is made audible by hearing aids or cochlear implants (CIs). While hearing loss can impact central auditory pathways, at least part of the problem lies in the degradation of the signal at the input (Moore, 2008, for review). This is particularly exacerbated in CIs, which do not transmit spectro-temporal fine structure information to the listener. As a result, CI users have significant deficits in pitch perception (e.g., Shannon, 1983; Zeng, 2002; Chatterjee and Peng, 2008; Kong and Carlyon, 2010), which is a dominant cue for monaural stream segregation in normally hearing (NH) listeners. To date, there are mixed findings regarding stream segregation in CI listeners and also whether aging might affect this ability. The primary aim of the current study was to elucidate the effect of spectral smearing on stream segregation using an objective task in a group of adult listeners across a broad age range.

### A. Sequential stream segregation

Auditory stream segregation, the process by which independent auditory streams are perceptually analyzed, may operate on simultaneous or sequential inputs. Sequential auditory stream segregation is observed in laboratory experiments in which the listener perceptually separates temporally interleaved “streams” of stimuli. When sequences of tone triplets or doublets—ABA or AB (A and B typically have different frequencies, *f*, with the difference denoted as *Δf*)—are presented under conditions favoring “perceptual integration” (e.g., slow presentation rate or small Δ*f*), listeners hear a single perceptual stream with a galloping rhythm (termed “temporal coherence”). When tones are presented under conditions favoring “perceptual segregation” (e.g., fast presentation rate or large Δ*f*), the galloping rhythm is lost, and two independent streams are clearly heard (termed “fission”). An insufficiently fast presentation rate or small *Δf* can result in a more ambiguous situation. Thus, within the same experimental session using identical parameters, users may perceive two distinct streams in some trials but not in others (Van Noorden, 1975). In the laboratory, listeners typically undergo training in distinguishing the two percepts (temporal coherence and fission) and, in the experimental paradigm, indicate which of the two they heard. The stimulus presentation rate and/or the frequency difference can be varied to alter the boundary between these two percepts (Van Noorden, 1975).

**FIG. 1.**
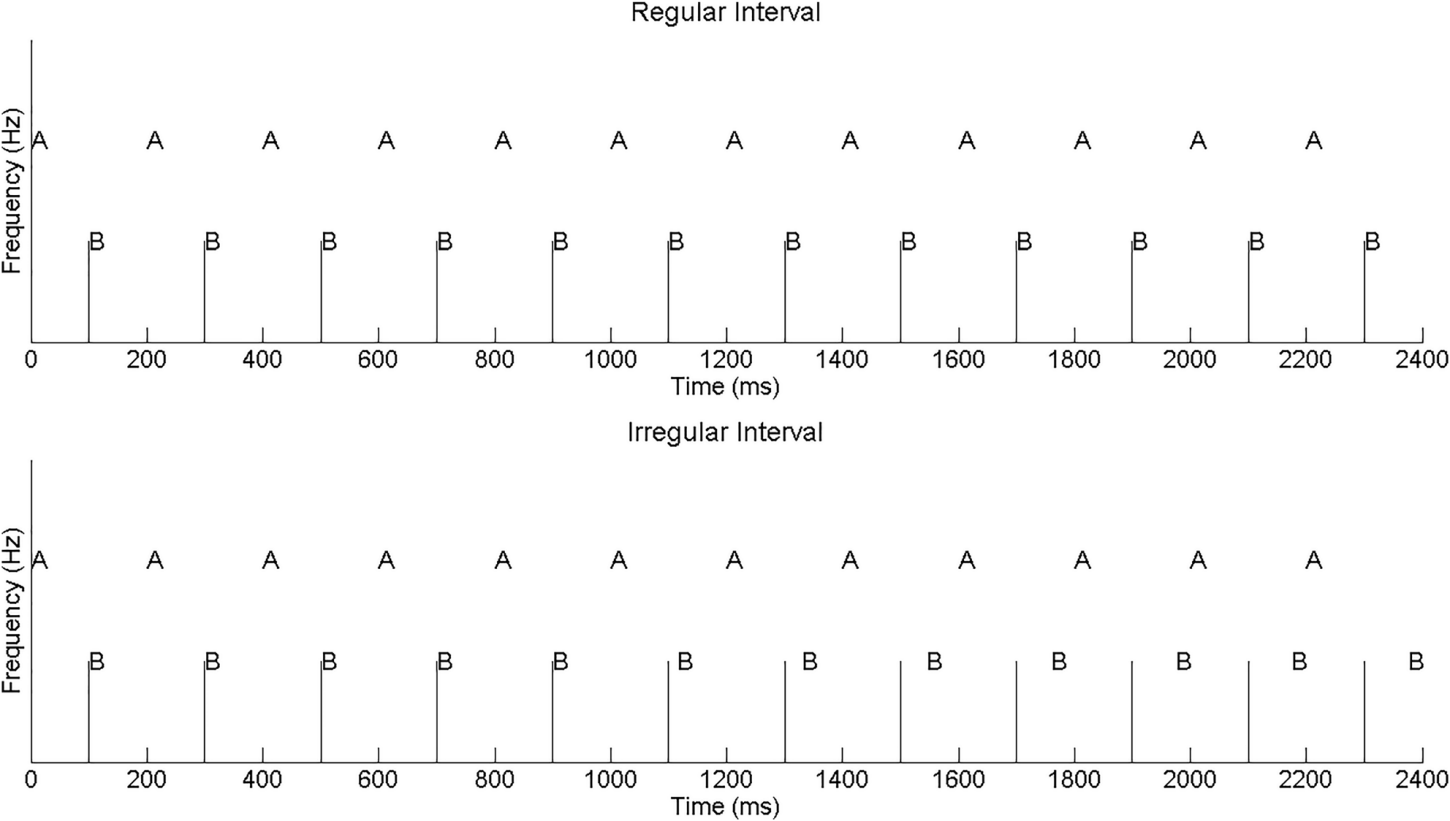
Experimental paradigm for AB streaming. The vertical bars are spaced at equal intervals of 200 ms. Note that for the irregular rhythm, the B stimulus is eventually presented at a delay (visualized as an offset from the vertical bar), which makes the rhythm non-isochronous.

A more objective method of measuring streaming ability involves a two-interval forced choice (2-IFC) irregular rhythm detection task (IRD), in which a temporally interleaved sequence of AB pairs is presented (Cusack and Roberts 2000). The sequence is isochronous in the reference interval (i.e., the timing and rhythm remain the same throughout the duration). In the experimental interval, the sequence is initially isochronous. Subsequently, the onset of B is gradually delayed, resulting in an irregular rhythm that is theoretically detectable if A and B fall in the same perceptual stream (Fig. 1). An important feature of this task is that the irregularity detection threshold (delay of B required to detect the irregularity) is *higher* when A and B fall in different perceptual streams. Thus, greater (and better) perceptual segregation is linked to poorer performance in the IRD task. Additionally, performance on the IRD task, as well as other stream segregation tasks, has been correlated to speech perception ability in CI users (Hong and Turner, 2006) and HI listeners (Mackersie *et al.*, 2001).

### B. Stream segregation in individuals with hearing loss and cochlear implants

It has been hypothesized that HI listeners or CI users might experience deficits in stream segregation abilities due to a loss of spectral and/or temporal resolution. However, results with HI listeners appear to be mixed: some studies have shown significant streaming deficits in HI listeners compared to NH listeners (Grimault *et al.*, 2001; Mackersie *et al.*, 2001), while others have reported no effect of hearing loss on streaming abilities (Rose and Moore, 1997; Valentine and Lentz, 2008).

Likewise, measures of stream segregation among CI listeners also yield conflicting results. Chatterjee *et al.* (2006) presented controlled pulse trains as ABA triplets directly to electrodes via a direct stimulation research interface and used a subjective rating method, while Hong and Turner (2006, 2009) presented pure tones and amplitude-modulated noisebands via speech processors in an IRD task. All three studies found evidence for stream segregation in some CI users, though large individual variations in performance were reported. Hong and Turner (2006) also observed that segregation ability was correlated with speech perception in noise and babble backgrounds. One disadvantage of using acoustic stimuli presented through a speech processor, as in Hong and Turner (2006, 2009), is that the processor alters the information transmitted to the listener. For example, the automatic gain control (AGC) cannot be readily controlled in the processors. The AGC algorithm considers characteristics of the input signal (e.g., signal level, envelope fluctuations) and modifies the gain and compression accordingly. The attack and release time characteristics of the AGC can have significant impact on the temporal and amplitude properties of stimuli such as sequences of brief tones and therefore can confound interpretation of results when performing an IRD task with stimuli presented in the soundfield.

Cooper and Roberts (2007, 2009) attempted more rigorous stimulus control by using an experimental speech processor in which a single electrode was activated at a time when acoustic pure tone stimuli were presented. The stimuli corresponded to the center frequency of the passband assigned to each electrode, based on the CI user's MAP. Using a subjective sequential streaming task, Cooper and Roberts (2007) did not see an effect of presentation rate on the ability to stream ABA triplets, and argued that CI users were using pitch discrimination to perform the task. Cooper and Roberts (2009) compared CI listeners' performance on streaming a long sequence of AB pairs and a single presentation of an ABA triplet. They found no interaction between electrode separation and sequence length and suggested that this reflected a lack of stream segregation, since electrode separation for longer sequences did not promote improved stream segregation as would be expected (Anstis and Saida, 1985; Cusack *et al.*, 2004; Roberts *et al.*, 2008).

As mentioned previously, pitch salience is a useful cue for both simultaneous and sequential stream segregation, both of which are likely involved in speech understanding in competing backgrounds. For instance, HI listeners who show difficulty with a fundamental frequency (*f*0) discrimination task also show greater difficulty on speech recognition in background noise (Summers and Leek, 1998). The representation of *f*0 is severely compromised in CIs (e.g., Qin and Oxenham, 2005; Chatterjee and Peng, 2008) due to limited frequency resolution. For CI listeners, electrode discrimination abilities, both in single and in multi-channel contexts, are key for pitch sensitivity. Given the importance of pitch and spectro-temporal fine structure in auditory scene analysis, reduced pitch perception in CI users may potentially affect both sequential and simultaneous stream segregation and, by extension, speech recognition in complex backgrounds.

There is a possibility that, when listeners complete the IRD task intended to measure stream segregation, they can instead attend to differences in the temporal gaps between the component stimuli. In other words, performance could reflect gap discrimination ability rather than stream segregation ability. In both CI and NH listeners, gap detection (and presumably gap discrimination) thresholds increase with increasing channel (or frequency) separation between the markers flanking the gaps (Chatterjee *et al.*, 1998; Formby *et al.*, 1998; Hanekom and Shannon, 1998). Similarly, performance on the IRD task worsens with greater frequency separation, which, as aforementioned, is interpreted as better stream segregation ability (Hong and Turner, 2006; Valentine and Lentz, 2008; Cooper and Roberts, 2009). It is possible that CI listeners, who might not in fact form separate perceptual streams, instead perform the task as a gap discrimination task even with large A-B differences. Prior studies have looked at possible relationships between gap detection and stream segregation ability in the same CI participants. A lack of correlation between performance measures on the two tasks would imply that the two measures reflect different perceptual phenomena. Hong and Turner (2006) argued that listeners could potentially perform the IRD task by paying attention to the end of the non-isochronous rhythm, where there is the greatest temporal delay in B relative to A. They compared the results of the IRD task to a short rhythm discrimination task in NH and CI listeners; these listeners had lower (better) thresholds for the short rhythm discrimination task compared to the IRD task. Duran (2010) and Duran *et al.* (2012) measured single channel stream segregation ability based on stimulation rate using the IRD paradigm, where pulse trains of different rates were presented to a single electrode using direct stimulation. Additionally, the CI listeners performed a single channel gap detection task, in which the markers were pulse trains of different rates (Duran, 2010). Similar to Hong and Turner (2006), performance in the IRD paradigm could not be accounted for by performance in the gap detection task (Duran, 2010). If indeed the IRD task is performed by CI listeners as a gap discrimination task, then their performance in the IRD task should be highly correlated with the discriminability of the component A and B stimuli. Thus, more discriminable stimuli would be associated with higher gap thresholds and therefore higher IRD thresholds. In the present study, a comparison of electrode discrimination with performance in the IRD task in the same listeners provided a direct test of this hypothesis.

Previous work on stream segregation by CI listeners suggests that some elements of perceptual segregation are present in some CI listeners. However, not all elements of stream segregation, including features such as “build up” of streaming (increasing separation of two perceptual streams over time; Anstis and Saida, 1985; Cusack *et al.*, 2004; Roberts *et al.*, 2008), the subjective experience of attentional switching from one stream to another, and the perceptual instability between integration and segregation (Anstis and Saida, 1985; Cooper and Roberts, 2007), have been shown in electric hearing. In addition, these aspects of stream segregation are often measured using more subjective techniques (some exceptions of interest are reported in Billig and Carlyon, 2016); although subjective reports have their own value in the stream-segregation literature, the present study was designed with a more objective measure in view. In addition, it may be difficult to train a CI user on a subjective stream segregation task if they truly cannot stream. It is possible that if some CI listeners are able to partially segregate streams to the extent that the IRD task reflects such segregation, then these listeners may be at an advantage in background noise (Hong and Turner, 2006), even if they are unable to fully segregate the streams as well as NH listeners can. Here, we take the approach that the IRD task reflects at least partial stream segregation, and focus on specific aspects of the process in NH and CI listeners. These include effects of frequency separation and reduced pitch salience as the stimuli change from pure tones to narrow-band noises in NH listeners, and effects of electrode separation in direct electrical stimulation, as revealed by CI and NH listeners' performance in this task. Throughout this paper, when we use the term “stream segregation” in the context of our results, we imply those aspects of stream segregation revealed by the IRD task.

### C. The role of aging in sequential stream segregation

The role of aging in stream segregation also remains unclear regardless of hearing status. Snyder and Alain (2006) observed no significant differences among younger, middle-aged, and older NH listeners (YNH, MNH, ONH) when measuring streaming abilities using pure tone ABA triplets and a subjective method. Older HI (OHI) listeners were able to stream harmonic complexes in an IRD task (Stainsby *et al.*, 2004), although there was no direct comparison to YNH. Mackersie *et al.* (2001), however, found that OHI listeners had significantly higher fusion thresholds (threshold frequency separation that leads to temporal coherence) than YNH listeners when measuring streaming abilities using pure tone ABA triplets and a subjective method. The thresholds for the HI listeners were negatively and significantly correlated with sentence recognition in a dual-talker task. In other words, higher fusion thresholds implied decreased streaming ability, which was correlated with a reduced ability to recognize the target sentence. Given that the study did not include age-matched NH listeners, it is not clear whether the higher fusion thresholds reflect declines in hearing sensitivity, aging effects, or both. Mixed results across these studies may also reflect the use of differing experimental paradigms—a subjective ABA streaming task was used in some cases while an objective IRD streaming task was used in other cases.

CI listeners who participate in perceptual experiments span a wide range of ages, and often such studies include a number of older CI listeners. As CI patients represent a small proportion of the population, they are difficult to recruit in sufficient numbers to allow for studies of aging effects. In particular, age effects may play a role in the rhythm-related tasks described here. To perform the IRD task, listeners compare the temporal gaps between successive A and B stimuli to determine if the rhythm is irregular. Age-related declines in timing-related and gap detection tasks have been demonstrated using other measures (e.g., Fitzgibbons and Gordon-Salant, 1994, 2004) and may influence IRD task performance in the present study. Older listeners who display rhythm detection difficulties may show elevated thresholds on the IRD task, which will overestimate their true stream segregation abilities. Therefore, the present study included both younger and older NH listeners who were similar in age range to the older CI participants.

### D. Objectives of the present study

We measured stream segregation in a group of CI listeners using both pure-tone stimuli presented via their everyday speech processors and electrical pulse trains presented via direct stimulation using a custom research interface. The IRD task was used in both cases. The objective of measuring stream segregation in these two ways was to allow a comparison of listeners' performance with the highly controlled pulse trains heard through our research interface, and the stimuli heard through their everyday speech processor. Factors that might contribute to observed differences include the filter widths of the speech processor, which would send small-amplitude pulse trains to neighboring electrodes other than the electrode receiving the bulk of the pure tone signal, AGC time constants, as well as the mapping of acoustic amplitude to electric current. In contrast, the research interface would send the pulse train to a single electrode, without any AGC intervening, and at carefully loudness-balanced current levels. In the same subjects, we also measured electrode discrimination ability using direct electrical stimulation to examine the role of place coding in stream segregation by CI listeners. The correlation between electrode discrimination and IRD thresholds was also interpreted as indicative of the possible use of gap discrimination rather than stream segregation in the IRD task.

To address the potential confounds introduced by age effects, we also measured stream segregation by a group of YNH and ONH listeners using both the same pure tones used with CI listeners and narrowband noises that were centered on the same frequencies as the pure tones and had reduced pitch salience. The purpose of the narrowband noises was not to directly simulate spectral smearing in cochlear implants, but rather to examine the effects of reduced pitch salience on YNH and ONH listeners' stream segregation abilities.

In summary, IRD task performance was measured using equal-rms pure tones (YNH, ONH, CI), equal-rms noisebands (YNH, ONH), and loudness-balanced, electrical stimuli presented via direct stimulation (CI). In addition, electrode discrimination ability was measured in the CI listeners using the same stimuli.

## II. METHODS

The study was conducted at the University of Maryland and received prior approval from the University of Maryland Institutional Review Board. Prior to the study, participants signed an informed consent form.

### A. Participants

Participants consisted of four YNH listeners (mean age: 19.75 yr, SD: 2.06 yr), four ONH listeners (mean age: 68.25 yr, SD: 4.57 yr), and six Cochlear Ltd. CI users (mean age: 55.66 yr, SD: 15.22 yr; see Table I). YNH listeners had audiometric thresholds ≤20 dB hearing level (HL) bilaterally for standard audiometric frequencies (250–8000 Hz). ONH participants had thresholds ≤25 dB HL bilaterally, except for one who had a 30 dB HL threshold (right ear, 6000 Hz) and a 35 dB HL threshold (bilaterally, 8000 Hz). CI users did not have residual hearing in their implanted ear. Bimodal users removed their contralateral hearing aid during testing.

**TABLE I.** Relevant information for CI participants.

Participant	Age	Gender	Onset of deafness	Implant (ear)	Processing strategy	Years of implant experience
CI 1	27	M	Prelingual	N-24 (Right) Freedom (Left)	ACE (N-24)	9 (N-24) 3 (Freedom)
CI 2	59	F	Perilingual	N-24 (Right)	ACE	11
CI 3	64	M	Postlingual	N-24 (Right)	ACE	6
CI 4	67	F	Postlingual	Freedom (Right)	ACE	3
CI 5	66	F	Postlingual	Freedom (Right)	ACE	2
CI 6	51	M	Postlingual	N-24 (Right)	ACE	9

### B. Acoustic stimuli

Stimuli were 60-ms pure tones and narrowband noises (NBN) of 1/2, 1/3, and 1/5 octave bandwidths. Center frequencies ranged from 793 to 5039 Hz spaced by 1/3 octave intervals. Stimuli were created with a 44 000 Hz sampling rate in matlab. They were filtered with a Blackman–Harris function and equalized in rms intensity using Adobe Audition 1.5. For each center frequency, five independent sets of 1/2, 1/3, and 1/5 octave bandwidth NBN and one set of pure tones were created. (Refer to the last paragraph of Sec. II C for the rationale for using independent sets of NBN stimuli.) To create the NBN stimuli, we first created five different NBN files with randomly fluctuating amplitudes drawn from a uniform distribution. Subsequently, for each of the five NBN files, Blackman–Harris filtering was applied with the appropriate cutoff frequencies. Thus, there were a total of nine pure tone files and 135 NBN files (five beginning noise files × 3 bandwidths × 9 frequencies).

### C. Stream segregation of acoustic stimuli

Experiments were conducted in a double-walled, sound-attenuating test booth. For NH listeners, stimuli were routed through an external Edirol UA-25 soundcard, a Rane SM 26B Splitter/Mixer, and presented monaurally to the listener's better ear via a Sennheiser HDA-200 supraural headphone at 65 dBA. The better ear was determined by a numerical average of audiometric thresholds from 250 to 8000 Hz. For CI listeners, stimuli were routed through a Crown D-75 A amplifier and presented at 65 dBA via a Tannoy Reveal speaker. Participants wore their CIs with their clinically assigned processors. CI1 only utilized his N-24 implant. Bimodal participants did not wear their contralateral hearing aid. Additionally, the stimuli were not sufficiently audible for the unaided contralateral ear to detect due to the severity of hearing loss in that ear.

A 2-IFC, two-down, one-up adaptive IRD task was used. In the reference interval, the A-to-A and A-to-B interstimulus intervals were fixed at 140 and 40 ms, respectively, resulting in an isochronous rhythm of 22 AB repetitions lasting 4.4 s. In the experimental interval, the sequence was presented isochronously for the first 2.4 s (12 repetitions). Next, the onset of B was increasingly delayed (following a linear ramp) for one second (five repetitions). This delay was fixed at the maximum delay for that interval for the final one second (five repetitions). The listener indicated which interval contained the irregular rhythm. The delay of B was increased for incorrect responses and vice-versa. The final delay threshold was based on six to eight reversal points. The initial step size (first four reversals) was 4 ms and the final step size was 2 ms.

Both intervals contained one of four possible AB sequence types (pure tones, 1/2, 1/3, or 1/5 octave bandwidth NBN); the presentation order of stimulus type was randomized. The center frequency of A was fixed at 2000 Hz, as this frequency should theoretically correspond to electrode 10 (E10) in the frequency-allocation tables for the CIs used. The B center frequency was randomly selected from the set of nine frequencies described previously, including 2000 Hz. Thus, there were nine conditions, corresponding to the nine center frequencies of B. Three repetitions per condition were performed.

In the intervals containing NBN, care was taken to ensure that inherent spectro-temporal fine structure fluctuations in successive A stimuli and successive B stimuli were not identical, as consistent fluctuations can be used as a cue for segregation (as observed in pilot data). As stated previously, five different versions of NBN were initially created, each with randomly fluctuating amplitudes drawn from a uniform distribution (see Sec. II B). Each A or B stimulus was randomly chosen from one of the five NBN stimuli of the same bandwidth. For example, a sequence could contain A_1_B_3_A_4_B_5_A_2_B_1._, where the A and B stimuli had fixed center frequencies but were randomly chosen from one of the five NBN stimuli (represented by the subscripts).

### D. Electric stimuli

Stimuli were loudness-balanced, 60-ms biphasic pulse trains (40 *μ*s/phase, 10 *μ*s phase delay) presented at a 1000 Hz pulse rate in MP1 mode. They were presented through a custom-built research interface [(HEINRI, House Ear Institute Nucleus Research Interface) Shannon *et al.*, 1990; Robert, 2002]. In this device, electrodes are numbered 1–22 from base to apex. Stimuli were presented to electrodes 2, 4, 6–14, 16, and 18.

Loudness-balancing was performed using a double-staircase, 2-IFC, two-down, one-up (descending track) and two-up, one-down (ascending track) adaptive method (Jesteadt, 1980; Zeng and Turner, 1991). In the reference interval, a pulse train was presented to electrode 10 at 50% of the user's dynamic range; the experimental interval contained a pulse train presented to electrodes 2–18. Participants listened to stimuli from both intervals and judged the loudness of the experimental stimuli relative to the reference stimuli. The experimental stimulus was judged louder than the reference 70.7% and 29.3% of the time, respectively, when the descending track and ascending track converged (after eight to ten reversals) (Levitt, 1971). The mean of the two levels at the end of the two tracks was based upon the final four to six reversal points, and was considered the loudness-balanced level. Initial step sizes (first four reversals) were 0.4 dB re: μA; final step sizes were 0.2 dB re: μA. Loudness-balancing was performed two to three times; the average of these runs was used as the loudness-balanced presentation level.

### E. Stream segregation of electric stimuli

All A stimuli were delivered to E10. The B stimuli were delivered to one of 13 possible electrodes (2, 4, 6–14, 16, and 18); the electrode utilized was randomized. Five repetitions for each of the 13 electrode conditions were performed. Due to loudness sensitivity issues, E2 was not used for CI 3, and E2 and E4 were not used for CI 4.

The identical, adaptive IRD task described previously was used for the electric stimuli. The only difference was the use of pulse trains, and the delivery of the pulse trains via the research interface.

### F. Electrode discrimination

In a 3-IFC task, two reference intervals and one experimental interval contained the aforementioned loudness-balanced pulse trains. The reference intervals were identical pulse trains presented to E10. The experimental interval (randomly, one of the three intervals) was a pulse train presented to electrodes 2, 4, 6–14, 16, and 18, thus giving 13 comparison conditions. The listener was asked to choose the interval that sounded different. The percentage of correctly chosen intervals out of 40 repetitions per condition was calculated.

### G. Electrical pulse trains presented via research interface vs acoustic tones presented via speech processor

**FIG. 2.**
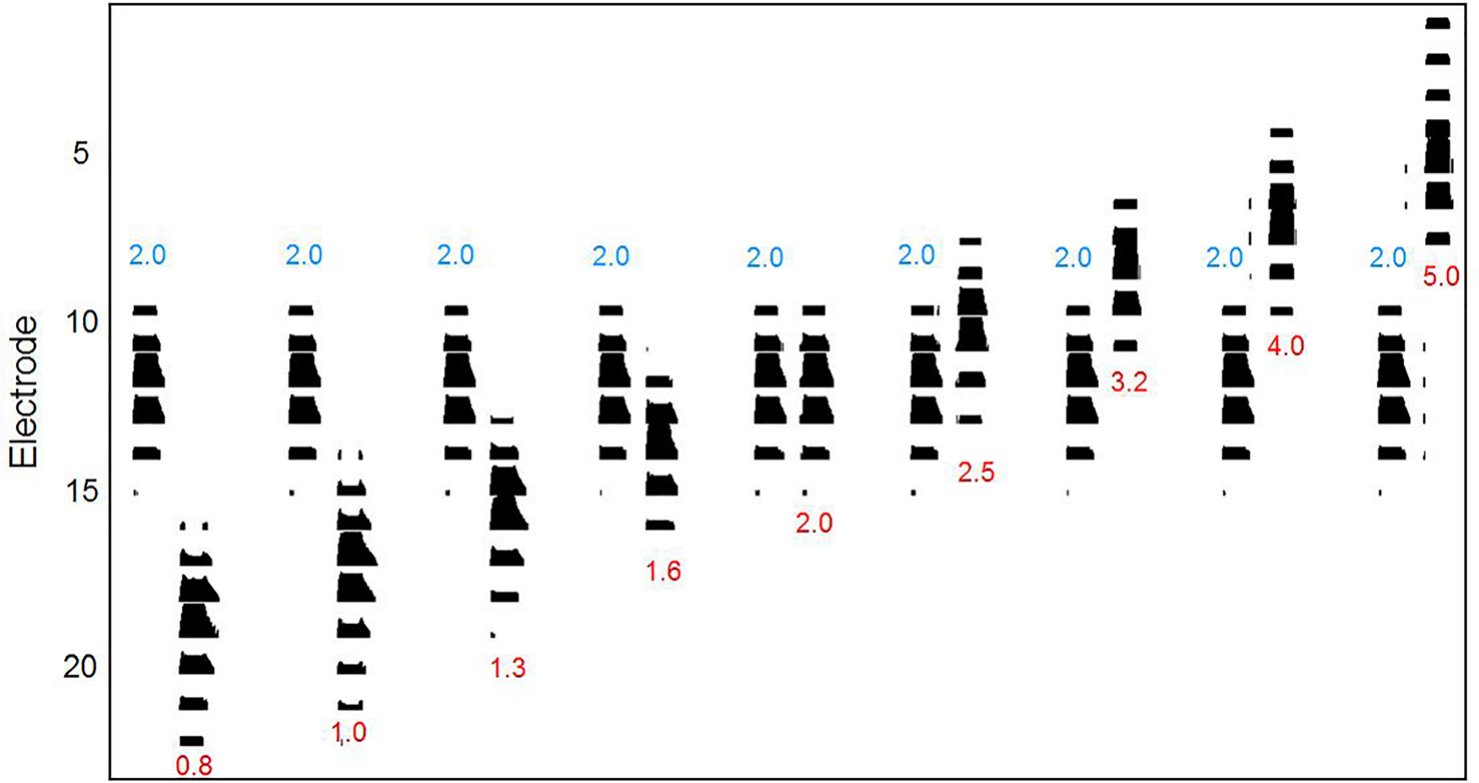
(Color online) Electrodogram of the stimulus pattern resulting from each pair of pure-tone AB stimuli, generated based on a default frequency allocation (188–7938 Hz), stimulation rate (900 Hz), and eight maxima. Frequency values are in kHz. The A stimuli are indicated by the 2 kHz label. The B stimuli are labeled by their frequencies (ranging from 0.8 to 5 kHz). The vertical axis shows the electrode number. Note that each pure tone results in the stimulation of eight electrodes, resulting in a broad excitation pattern across the cochlea.

The target electrode for the pulse trains presented through the research interface, E10, was selected because it fell approximately in the region of the electrode array receiving acoustic information in the 1800–2300 Hz band for the speech processor settings of the participants in this study. Given the frequency-to-electrode maps in their speech processor settings, we would expect the 2000 Hz acoustic tones used in this study to generate stimulation primarily in E11 and E10, with minor variations across subjects. These variations were caused by the specific frequency map used to program the speech processor, which differs if electrodes are deactivated. Across subjects, E10 received information from a mean center frequency of 2139 Hz (SD 136 Hz) based on the average center frequency to electrode to assignment for each subject's unique speech processor map; likewise, the mean bandwidth of the filter assigned to E10 was 284.3 Hz (SD 49.3 Hz). Note that the neural populations excited by these electrodes are expected to be highly overlapping, so mechanisms of stream segregation for the acoustic tones are unlikely to be strictly influenced by exactly which electrodes received the stimulation. Figure 2 depicts an electrodogram of the stimulation pattern resulting from the stimuli, assuming a default frequency allocation (188–7938 Hz), stimulation rate (900 Hz), and eight maxima. As expected, the location of overlap varies based on the frequency of the A and B stimuli. In the figure, the A stimulus (the 2 kHz tone) excites eight electrodes, and each of the B stimuli excites eight electrodes. The large number of electrodes stimulated by each of these stimuli means that specificity is largely lost: the discriminability of these stimuli is likely to be strongly impacted by the broad neural excitation pattern that would result from such broad stimulation patterns. In contrast, direct electrical stimulation via the research interfaces allows us to selectively stimulate one electrode at a time.

Another minor difference between the two types of stimulation (acoustic versus electric in CI) was the stimulation mode. For technical reasons, direct stimulation through the interface was only possible in MP1 mode, whereas the default mode for the subjects' speech processors was MP1+2. Both should create broad excitation patterns.

Finally, the speech processor stimulation rates varied across subjects. All subjects used the advanced combination encoder (ACE) processing strategy, with per-channel pulse rates of either 720 or 900 pps (except for subject CI1 who used a 250 pps rate). On the other hand, the HEINRI was used to present stimuli with identical, fixed parameters to all subjects.

## III. RESULTS

### A. IRD with acoustic stimuli

#### 1. Normal hearing listeners

**FIG. 3.**
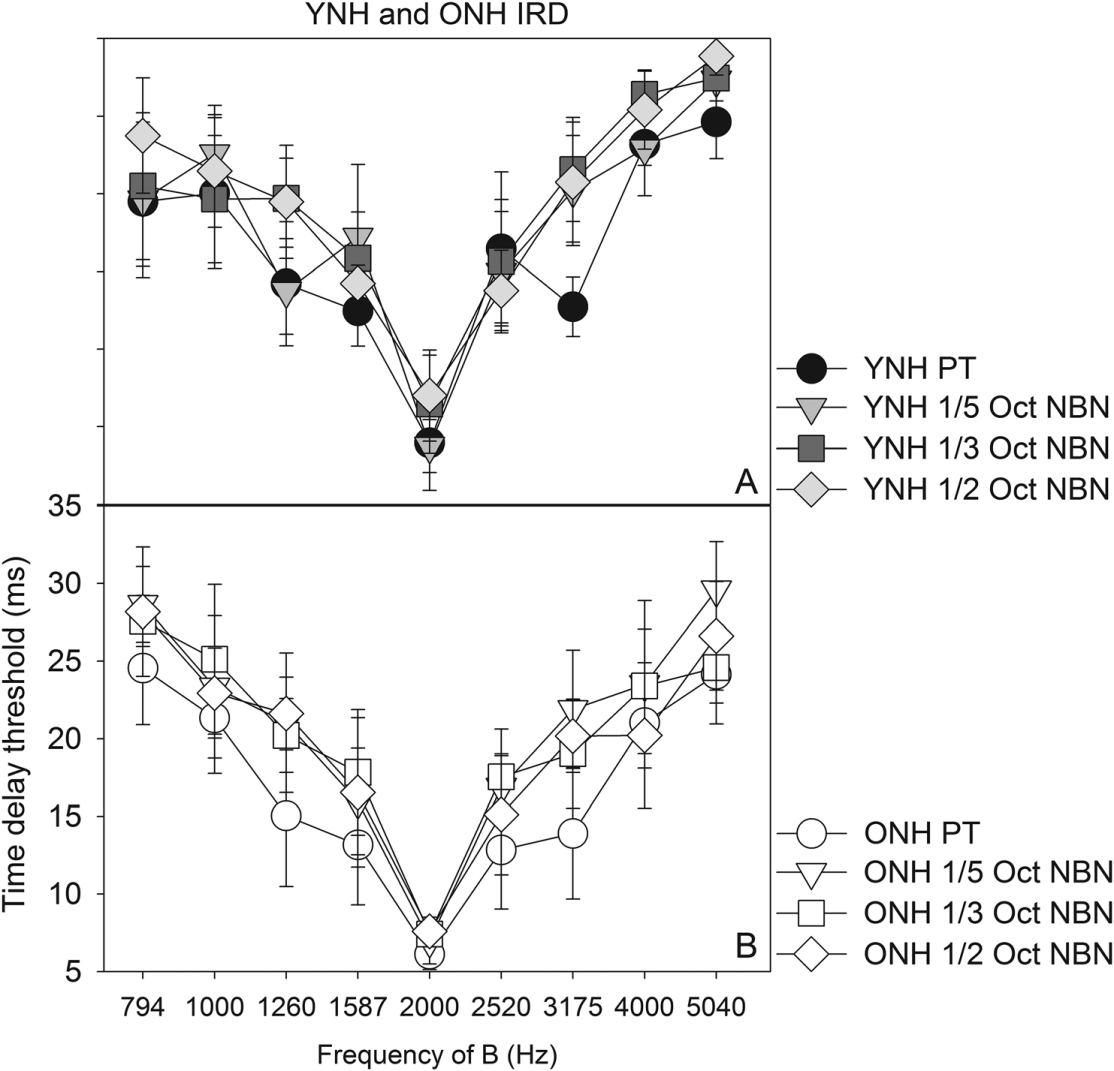
Average performance of YNH (A, top) and ONH listeners (B, bottom). Error bars show ±1 SEM.

Average IRD thresholds for YNH and ONH listeners are shown in Figs. 3(A) and 3(B) for the pure tones and narrow-band noises presented through headphones. Higher delay thresholds represent better streaming.

**FIG. 4.**
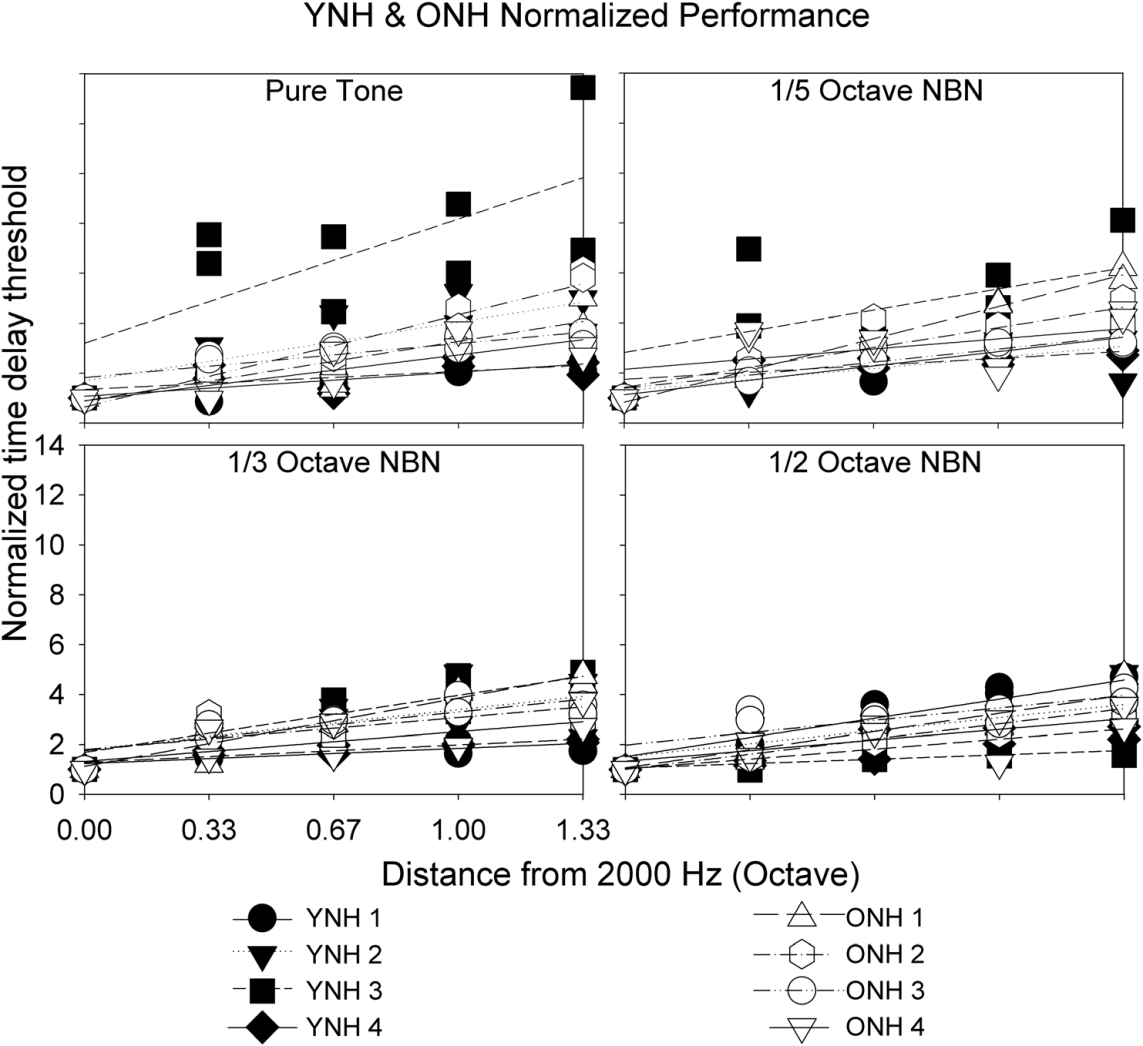
Individual normalized data obtained in YNH (filled symbols) and ONH (open symbols) listeners for all stimulus conditions, obtained by dividing threshold at each B frequency by threshold at B = 2000 Hz. Slopes of linear fits represent degree of streaming, with higher slopes interpreted as better stream segregation.

To compare the performance between YNH and ONH listeners, the threshold at each B frequency was divided by threshold at B = 2000 Hz to obtain individualized normalized thresholds. Slopes were obtained from linear fits of normalized threshold vs B frequency plots (Fig. 4, Table II). Filled and open symbols represent YNH and ONH performance, respectively, with higher slopes representing better stream segregation. Note that some YNH and ONH listeners occasionally could not detect the irregular rhythm despite introducing the maximum delay possible (39 ms—higher delays would cause temporal overlap between stimuli). Such data were not used in data analysis. In later analyses, we treated the CI data similarly. Additionally, Peirce's criterion was used to discard raw thresholds that were outliers (Peirce, 1852; Ross, 2003).

**TABLE II.** Slopes, *r*^2^ and significance values of linear fits of normalized thresholds.

	Pure tone			1/5 Octave NBN			1/3 Octave NBN			1/2 Octave NBN		
Participant	Slope	*r^2^*	*p*	Slope	*r^2^*	*p*	Slope	*r^2^*	*p*	Slope	*r^2^*	*p*
YNH 1	0.970	0.661	0.008*	1.726	0.813	0.002*	0.613	0.673	0.007*	2.298	0.800	0.001*
YNH 2	2.361	0.633	0.010*	1.338	0.400	0.067	1.602	0.399	0.068	1.559	0.496	0.034*
YNH 3	4.978	0.482	0.038*	2.534	0.321	0.112	2.277	0.770	0.002*	0.528	0.662	0.008*
YNH 4	0.724	0.330	0.106	0.839	0.499	0.034*	0.700	0.793	0.001*	1.194	0.664	0.007*
Avg ± SD	2.259 ± 1.951			1.609 ± 0.716			1.298 ± 0.791			1.395 ± 0.738		
ONH 1	2.393	0.704	0.005*	3.821	0.971	<0.001*	2.730	0.872	0.000*	2.181	0.791	0.001*
ONH 2	3.698	0.908	<0.001*	2.394	0.801	0.001*	1.278	0.537	0.025*	1.820	0.867	0.000*
ONH 3	1.367	0.689	0.006*	1.569	0.831	0.001*	1.540	0.699	0.005*	1.481	0.592	0.015*
ONH 4	1.839	0.670	0.007*	1.221	0.250	0.171	1.184	0.418	0.060	1.269	0.468	0.042*
Avg ± SD	2.324 ± 1.007			2.251 ± 1.156			1.683 ± 0.714			1.688 ± 0.400		

The Shapiro–Wilk test (Shapiro and Wilk, 1965) performed on the slope data indicated that the assumption of normality was not violated. A repeated-measures analysis of variance (ANOVA), with degree of spectral degradation as the within-subjects factor and age as the between-subjects factor, indicated no main effects of age (F_1,6_ = 0.122, p = 0.739) and degree of spectral degradation (F_3,18_ = 2.173, p = 0.127) on streaming performance. No interactions were observed (F_3,18_ = 0.127, p = 0.848). The lack of an aging effect is consistent with previous investigations by Snyder and Alain (2006) and Stainsby *et al.* (2004) in which ONH and OHI displayed streaming ability. However, both studies used tonal stimuli or harmonic complexes, preserving spectro-temporal fine structure. It appears that diminished spectral resolution did not affect stream segregation by the NH listeners in the present study. Given the lack of an age-related difference, the YNH and ONH data were pooled together in the remaining analyses and plots.

**FIG. 5.**
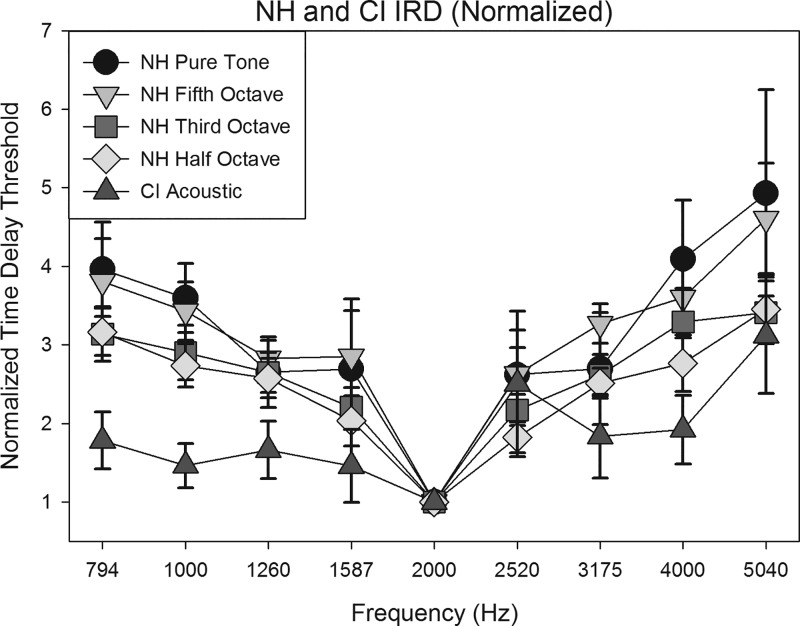
Average normalized performance for NH and CI groups for pure tone (NH, CI) and narrowband (NH) groups.

There also appears to be an effect of B frequency on stream segregation for the NH listeners (Fig. 3). That is, for each of the conditions (pure tone, 1/5 octave NBN, 1/3 octave NBN, and 1/2 octave NBN), greater separation between A and B frequency led to improved stream segregation. The normalized ONH and YNH threshold data were combined into a larger data set (Fig. 5). Separate one-way repeated measures ANOVAs (with Bonferroni corrections for follow-up pairwise comparisons) were performed for each of the four conditions. ANOVA results showed significant effects for each of the conditions (Table III), requiring follow-up pairwise comparisons to see where the greatest differences lie. Since 2000 Hz is the base frequency, we focused on the results of pairwise comparisons of threshold at B = 2000 Hz compared to all other B frequencies. In each condition, there were significant differences for most B frequencies, more so as the frequency separation increased. These results are shown in Table IV.

**TABLE III.** Results of one-way repeated ANOVAs performed on the datasets plotted in Fig. 5, showing significant effects of B frequency for NH listeners.

Condition	F (Greenhouse–Geisser)	P
NH pure tone	F_1.721,12.050_ = 7.436	0.010
NH fifth octave	F_3.092,18.552_ = 7.766	0.001
NH third octave	F_3.224, 22.565_ = 11.768	<0.001
NH half octave	F_3.904, 27.330_ = 13.3	< 0.001
CI pure tone	F_1.519, 7.597_ = 1.973	0.204

**TABLE IV.** Follow-up comparisons for ANOVAs done on the NH dataset (Table III) for each condition, indicating that greater frequency separations led to significantly higher thresholds (better streaming) in most cases.

	Condition			
B frequency (Hz)	Pure tone	Fifth octave	Third octave	Half octave
793	0.018*	0.018*	< 0.001*	<0.001*
1000	0.009*	0.009*	0.009*	< 0.001*
1259	0.072	<0.001*	<0.001*	<0.001*
1587	0.495	0.396	0.018*	0.135
2519	0.216	0.027*	0.009*	0.036*
3174	0.432	<0.001*	<0.001*	<0.001*
4000	0.036*	0.018*	0.009*	0.018*
5039	0.18	0.018*	0.009*	0.009*

#### 2. Cochlear implant listeners

**FIG. 6.**
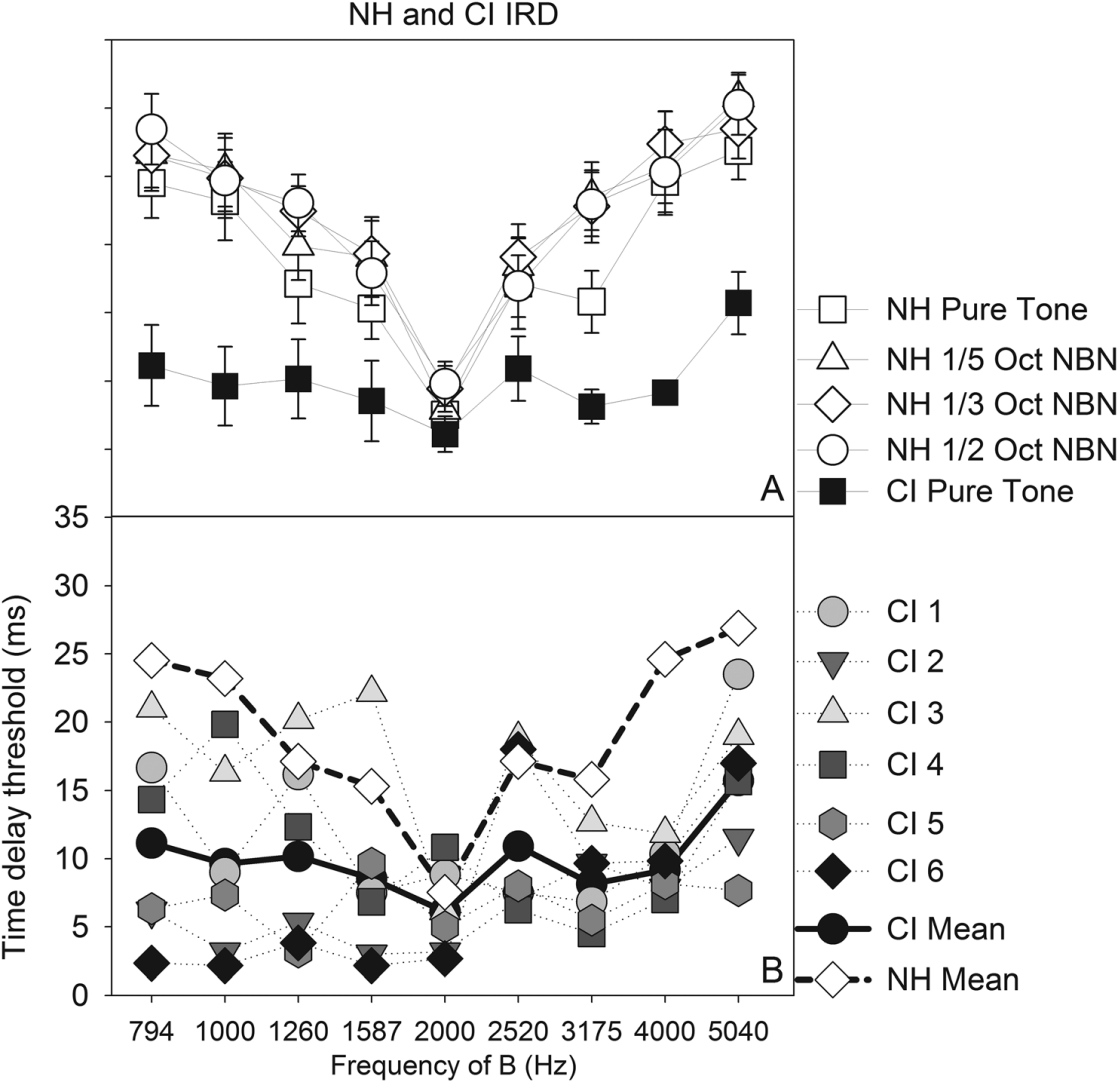
(A, top) Average performance of NH and CI listeners with acoustic stimuli. (B, bottom). Individual and average CI performance and average NH performance on streaming of pure tones.

In contrast to the results obtained with the NH group, CI listeners showed remarkable individual variability in IRD task performance with acoustic stimuli heard through their speech processor. Additionally, visual inspection suggests that, on average, CI users displayed poorer streaming of pure tones than NH listeners (Fig. 6). Strong intersubject variability has also been documented in previous studies of pure tone stream segregation by CI listeners (Hong and Turner, 2006; Cooper and Roberts, 2007, 2009). The CI listeners' data also varied less systematically with frequency separation than the NH listeners' data, making it difficult to calculate linear regression slopes. To quantitatively compare CI listeners' performance with NH listeners' performance, we performed a linear mixed effects analysis on the raw IRD thresholds, with subject group (NH, CI) and pure tone frequency as the fixed effects and subject-based random intercepts. Mixed effects modeling was particularly appealing because of the strong intersubject variability in the CI data set. Inclusion of subject-based random slopes did not improve the model. Results showed significant effects of subject group (F_1,12_ = 16.70, p = 0.0015) and pure tone frequency (F_8,96_ = 2.54, p = 0.0148), and a significant interaction between the two (F_8,96_ = 5.174, p = 0.0003). *Post hoc* Tukey contrasts on the effect of frequency showed significantly higher IRD thresholds (i.e., better stream segregation) when the B tone was at 5039.68 Hz vs at 3174.80 Hz (p = 0.034) and 2000 Hz (p = 0.001) and marginally significantly higher IRD threshold when the B tone was at 5039.68 Hz than at 1587.40 Hz (p = 0.059). *Post hoc* Tukey contrasts on the interaction between the subject group and pure tone frequencies showed that the groups were significantly different at the two lowest frequencies and the two highest frequencies of the B tone. On the low frequency side of the reference tone, IRD thresholds were significantly higher (i.e., better stream segregation) in NH listeners than in CI listeners at 793.7 Hz (p < 0.01) and 1000 Hz (p < 0.01). There were no other significant differences, and the IRD thresholds at 2000 Hz were also not significantly different between the groups. On the high frequency side of the reference tone, IRD thresholds were significantly higher in NH than CI listeners at 4000 Hz (p < 0.01) and marginally higher at 5039.68 Hz (p = 0.05). There were no other significant differences between the groups.

### B. Comparison of results obtained with electric and acoustic stimuli: CI listeners

**FIG. 7.**
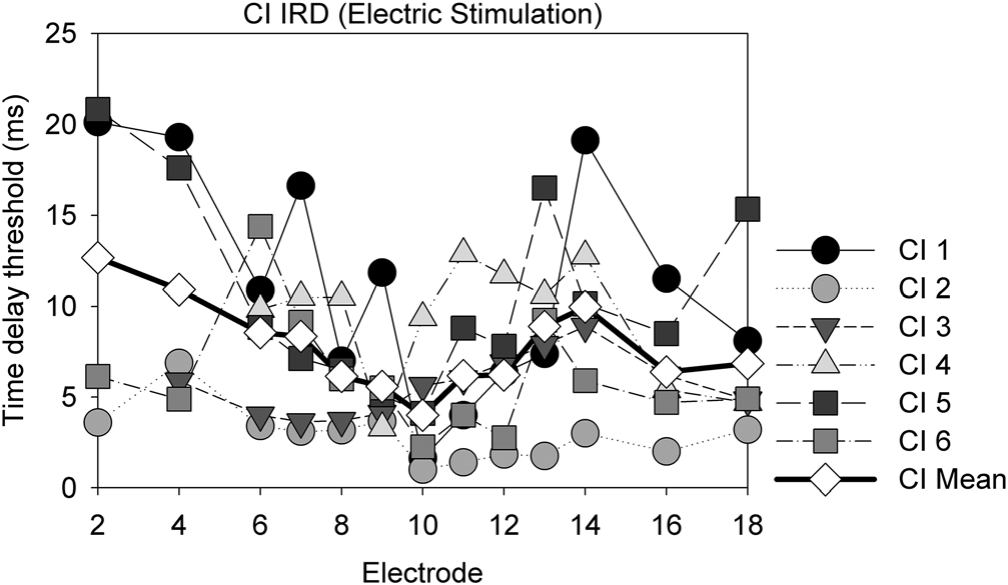
Average and individual performance of CI listeners with electric stimuli in the IRD task.

**FIG. 8.**
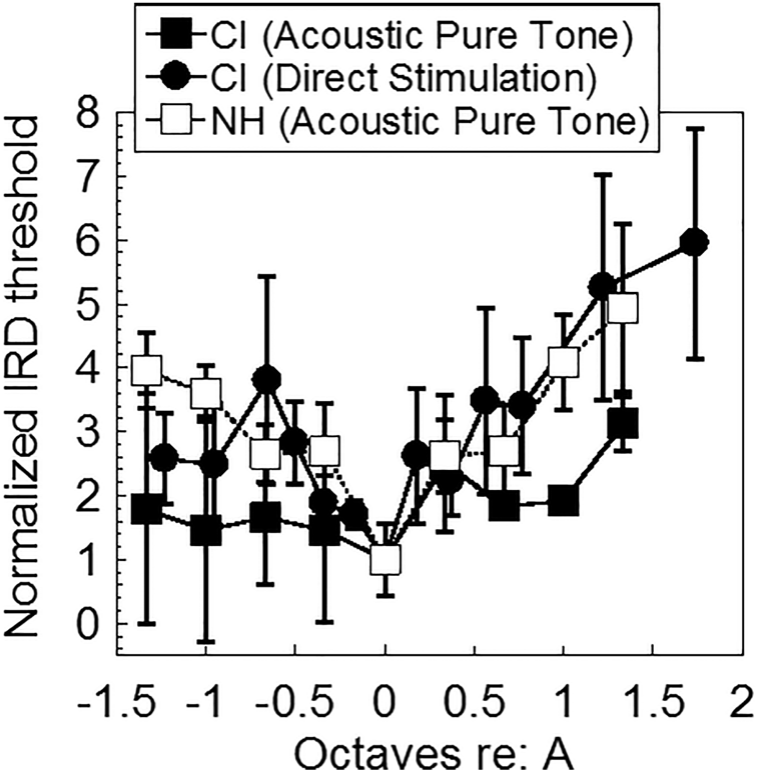
CI performance on streaming using both acoustic and electric stimuli, as well as NH performance. Both the frequency scale and the IRD threshold scale are normalized as described in the text. Error bars show ±1 SEM.

The CI listeners' IRD thresholds obtained with direct electric stimulation via the research interface showed considerable variation as well (Fig. 7), with some subjects showing little to no stream segregation and others showing considerable segregation. As the electrical pulse trains were applied to individual electrodes along the array, they were converted to approximate place-based pure tone frequencies using the Greenwood function (Greenwood, 1990) with a correction accounting for peri-modiolar positioning of the electrode array (Stakhovskaya *et al.*, 2007) to allow for comparison with acoustic stimuli. Estimation of electrode location was based on Contour dimensions provided by Cochlear Ltd. A standard electrode insertion and a cochlear length of 35 mm were used to approximate the corresponding frequency place for each electrode. E2-E18 approximates a range of 1143–8939 Hz (Greenwood, 1990; Stakhovskaya *et al.*, 2007), and E10, the reference “A” electrode used in the electrical stimuli, corresponds to 2687.25 Hz approximately. The frequency axis (for both pure tone and electric stimulation) was then normalized by converting each frequency to octaves re: the reference frequency (the reference frequency was 2000 Hz for acoustic stimuli and 2687.25 Hz for electrical stimuli). The IRD threshold values were normalized to the reference value obtained when B = A. Figure 8 plots the normalized CI data for comparison, in addition to NH pure tone data. Note that the pattern corresponding to direct electrical stimulation is the mirror image of that in Fig. 7, as the abscissa has been converted to a frequency scale rather than electrode number (Greenwood, 1990; Stakhovskaya *et al.*, 2007). In Fig. 7, the *x* axis starts from the basal end while in Fig. 8, the *x* axis starts from the apical end.

A linear mixed effects analysis was conducted on the normalized IRD thresholds for the CI users, with stimulation type (acoustic vs direct stimulation) and relative frequency of the B stimulus (in octaves re: A) as fixed effects and subject-based random intercepts. The model showed no improvement when subject-based random slopes were allowed. Results showed significant effects of relative frequency (F_1,120_ = 9.319, p = 0.003) and stimulation type (F_1,120_ = 9.02, p = 0.0033), with direct electrical stimuli yielding significantly better stream segregation than acoustic pure tones. There were no interactions. In a second analysis, the 0 octaves condition was removed and the basal-apical asymmetry in the data were examined by repeating the LME analysis, with stimulation type, relative frequency of the B stimulus (in absolute octaves re: A), and location of the B stimulus (basal or apical to A) as the fixed effects, and subject-based random intercepts. Results showed significant effects of relative frequency (F_1, 108_ = 8.53, p = 0.004), stimulation type (F_1,108_ = 12.99, p = 0.0005) and location (F_1,108_ = 6.14, p = 0.015), with basal locations showing significantly greater stream segregation than apical locations overall. Again, no interactions were found.

Figure 8 also shows a comparison of the normalized CI data (acoustic and direct stim) with the normalized data obtained in NH listeners attending to acoustic pure tones from Fig. 5. The NH data are surprisingly similar to the mean CI data under direct electric stimulation, although the CI data were more variable. An LME analysis conducted on the NH (pure tone) and CI (direct stimulation) data with frequency separation (octaves) and subject group as fixed effects, and subject-based random intercepts, confirmed the visual impression. There was a significant effect of frequency separation in octaves (F_1, 131_ = 9.63, p = 0.0023) but no significant effect of subject group and no interactions. The lack of a subject group effect appears to support the similarity between the NH (acoustic) and CI direct stimulation groups. However, we note that the lack of a statistical difference does not prove similarity, particularly given the large intersubject variability in the CI population, discussed in the next paragraph.

**FIG. 9.**
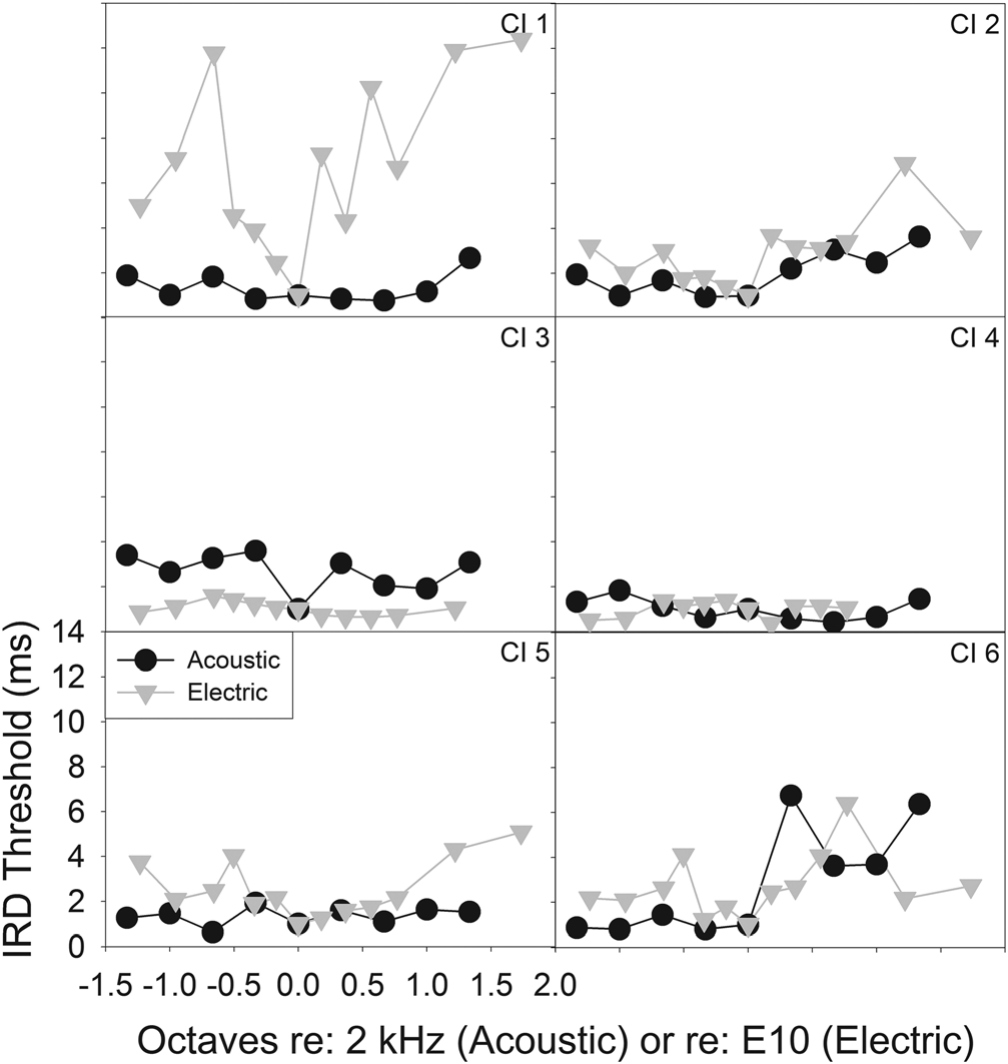
Individual normalized performance on streaming of acoustic and electric stimuli. Note that for most participants (except for CI 3 and 4), performance is better with direct stimulation.

To further examine the differences in performance when subjects listened to the soundfield acoustic and direct pulse train stimuli, we plot the individual performances (Fig. 9). Though across-electrode and across-subject variations in performance do exist, it appears that most of the participants (except for CI 3) display similar or improved streaming ability when using direct electrical stimulation. This underscores the possible effects that signal processing algorithms can have on the incoming acoustic stimuli. That is, some of the streaming deficits experienced by CI users in everyday listening may not just be attributed to reduced spectral/temporal resolution in the deafened auditory system—rather, perhaps the spectral and temporal cues of the incoming signal are further modified by the signal processing algorithm before reaching the auditory periphery.

### C. IRD and electrode discrimination with electric stimuli

**FIG. 10.**
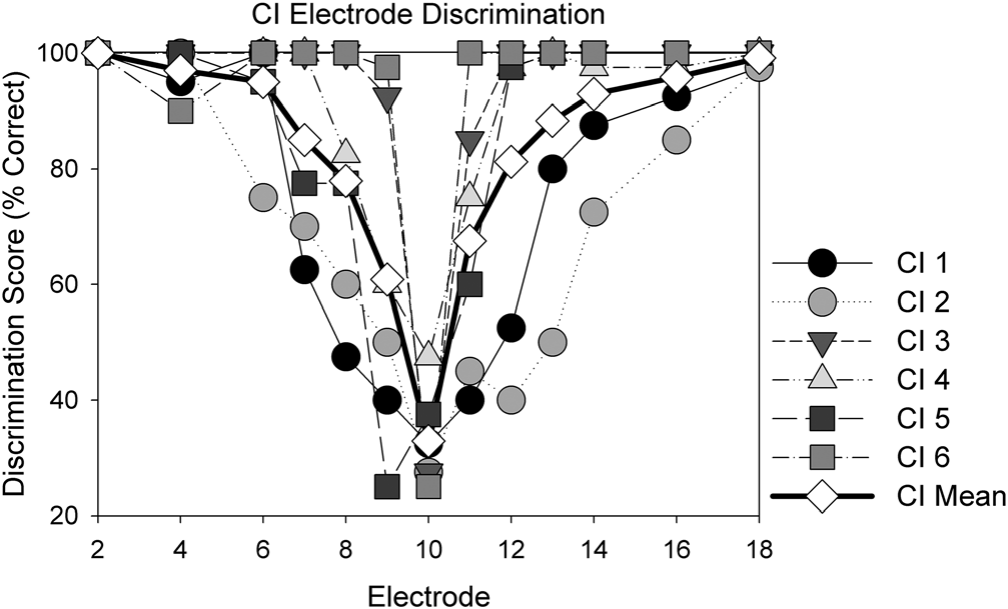
Average and individual performance of CI listeners with electric stimuli on electrode discrimination.

**FIG. 11.**
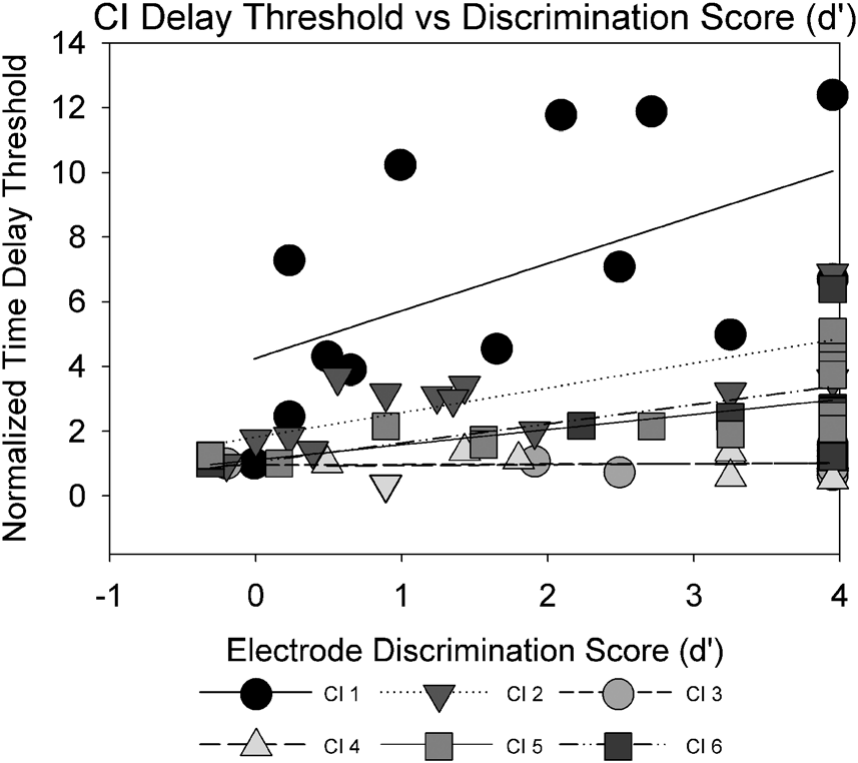
Normalized time delay thresholds plotted against electrode discrimination performance (d′ units) by the same CI listeners.

Results obtained in the electrode discrimination task suggested that CI users generally were able to discriminate among electrodes above chance (33.33% correct) beyond a two-electrode place separation (Fig. 10). As stream segregation and electrode discrimination both require electrode- or place-pitch cues, the relation between the normalized time delay thresholds obtained in the stream segregation task and electrode discrimination scores was examined. The electrode discrimination scores were converted to d′ values, and linear regression showed strong intersubject variability (Fig. 11). Scores of 100% correct were arbitrarily set to d′ values of 3.95. Steeper slopes of the linear regression (straight lines) were interpreted as suggesting increased availability of place cues for the streaming tasks. Participants CI1, CI2, and CI5 displayed steeper, statistically significant, slopes (0.59–1.46, p < 0.05) while the remaining participants displayed shallower, non-significant, slopes (0.02–0.47, 0.17 ≤ p ≤ 0.84). The steeper slopes for CI1 and CI5 may reflect increased salience of cues that improved streaming ability compared to remaining CI participants. An overall LME analysis with subject-based random effects showed no significant relation between electrode discrimination (d′) and IRD thresholds. The analysis was repeated after excluding the d′ values corresponding to 100% correct (which might have skewed the distribution of the data), and again showed no significant relationship. The negative finding also suggests that the stream segregation task did not reflect gap detection/discrimination ability.

### D. IRD raw threshold vs IRD slope with electric stimuli

IRD performance when A and B are the same electrode is effectively a within-channel discrimination task. If those results are correlated with stream segregation ability (as measured by the IRD slopes–e.g., Fig. 4) in the CI users, then it would suggest that the IRD task was reflecting electrode discrimination and not truly stream segregation. Thus, the raw threshold where A = B was compared to the IRD slopes for the CI data. No significant correlation was found in this case (r^2 ^= 0.3777, p = 0.194), consistent with the idea that performance in the IRD paradigm reflects the extent to which listeners experience stream segregation.

### E. IRD and temporal processing ability

To rule out temporal processing effects on IRD performance, raw delay thresholds obtained in the condition where stimuli A and B were both 2000 Hz were compared among YNH, ONH, and CI groups. This condition reduces to a simple rhythm discrimination task. The Shapiro-Wilk test (Shapiro and Wilk, 1965) performed on raw threshold data suggested that the assumption of normality was not violated for pure tone, 1/5, and 1/3 octave NBN data. The assumption was violated for 1/2 octave NBN data. A one-way ANOVA revealed no significant differences in raw thresholds among YNH, ONH, and CI groups for pure tones (F_2,11_ = 0.731, p = 0.504). Independent samples t-tests revealed no significant differences among YNH and ONH groups for 1/5 and 1/3 NBN (t_6_ = 0.769, p = 0.471 and t_6_ = 0.096, p = 0.108, respectively) while the Mann–Whitney U test revealed no significant differences among YNH and ONH groups for 1/2 octave NBN (U = 6, p = 0.561). The lack of significant differences suggests that known age-or hearing-status-related declines in temporal processing ability did not affect IRD task performance.

## IV. DISCUSSION

The NH participants showed strong resilience to reduced pitch salience from tonal to NBN stimuli. Even though the bandwidths of the NBN stimuli were wider than the corresponding auditory filters, the frequency separation between A and B stimuli appeared sufficient to overcome the spectrally smeared representation (Fig. 3; Table II). Bregman *et al.* (2001) also found that frequency separation in semitones, not bandwidth, was a significant predictor of NBN streaming ability in NH listeners. Similarly, we did find that as frequency separation increases, there was significantly more streaming (Table IV).

The results of the present study further suggest that NBN segregation ability is also not affected by age, as both YNH and ONH listeners demonstrated similar changes in stream segregation in all conditions as frequency separation increased (Fig. 3). There were no statistically significant differences between results obtained in YNH and ONH listeners, as demonstrated by slope data (Fig. 4). This indicates that noisebands up to half an octave in width retain sufficient perceptual differences across center frequencies to allow for stream segregation as measured in this task, and these perceptual differences can be utilized by both YNH and ONH listeners alike. These results also lend support to the findings of Snyder and Alain (2006), who also observed no significant aging effects. Additionally, they argued that concurrent, rather than sequential, stream segregation more readily declines in ONH listeners. For example, studies have demonstrated age-related declines in vowel identification during a dual-vowel presentation task (Summers and Leeks, 1998; Snyder and Alain, 2005), as well as age-related declines in the ability to detect a mistuned harmonic (Alain *et al.*, 2001).

In both acoustic and electric simulation, CI listeners showed improvements in streaming ability with increasing frequency difference, though nonmonotonicities were sometimes evident. CI listeners showed significantly poorer stream segregation than NH listeners when listening to pure tones through their speech processors. Certainly peripheral channeling cues play a role in stream segregation ability (Hartmann and Johnson, 1991), given that wider frequency separation led to improved stream segregation ability in the NH group and not the CI group for pure tone stimulation. Greater spatial/spectral interactions in the CI users likely contribute to the poorer performance despite the increasing frequency separation in the stimuli. However, the lack of fine structure information may also contribute to the poor performance in the CI group, given that within the NH group, there appeared to be a trend of poorer stream segregation ability as the bandwidth of the stimuli increased (Fig. 5), though this was a non-significant finding when looking at the slope data (Table II).

On the other hand, performance with direct electrical stimulation showed stronger evidence of stream segregation. Although there was considerable variability among CI patients, the normalized data in fact showed surprising similarities between the mean NH and mean CI results. LME analysis of the normalized NH (pure tone) and CI (direct stimulation) supported the observed similarity by showing a lack of a significant group effect (Fig. 8). However, we note the higher variance in CI results compared to the NH results as well as the range of individual stream segregation abilities amongst CI listeners. When looking at individual data for direct stimulation (Fig. 7), some CI users displayed very poor stream segregation ability (e.g., CI 3 and 4), while others displayed a pattern reminiscent of NH listeners (e.g., CI 1).

These differences between direct electric stimulation and acoustic stimulation likely arose from specifics of the speech processor algorithms. For instance, a pure tone processed through speech processor filters might stimulate multiple electrodes. In addition, the AGC mechanism might have caused significant temporal distortion of the stimuli. Further, amplitude mapping of CI processors is done with multi-channel stimulation in view, and maximum comfortable levels are reduced on a channel-by-channel basis to accommodate loudness summation. Thus, if the pure tones had stimulated one electrode at a time, the stimulus level of 65 dBA on a single tone might have resulted in a relatively soft sound. However, the pure tones actually stimulated eight electrodes at a time, which likely resulted in sufficient loudness summation for the stimuli to be reasonably loud. Unfortunately, we were not able to directly compare the loudness of the acoustic and electric stimuli in this study. With the research interface, more rigorous stimulus control was maintained and electric stimuli with defined parameters were delivered to one specified electrode. This likely resulted in more salient perceptual cues for stream segregation. Even with this added control, across-electrode variations were still likely present, reflecting additional factors beyond experimental control, such as neural survival, neural channel interactions, and more central processes involved in stream segregation which might be altered differently in different patients after deafness. For example, CI1, CI5, and CI6 presented a nonmonotonic pattern in streaming ability as electrode separation increased. Dips in streaming performance may reflect greater channel interactions (reduced spatial resolution and increased perceptual similarity) between E10 and the corresponding electrode where the dip occurred. For example, an E6–E10 channel interaction may account for the dip in CI1's performance at E6.

Few studies have directly compared NH and CI performance on streaming (e.g., Hong and Turner, 2006, 2009). Similar to our study, there were individual variations in CI performance in Hong and Turner's studies, which can affect conclusions regarding streaming ability. Using the AB paradigm and pure tone stimuli, Hong and Turner (2006) found that some CI users performed as well as NH listeners, while others performed worse. We infer that the poorer performance of the CI listeners in the present study cannot be easily explained by age-related effects, as the ONH group showed considerable robustness in stream segregation even when pitch cues were weakened.

Performance on the electrode discrimination task appeared to have little to do with CI listeners' stream segregation as measured in the present task. This apparent discrepancy suggests that the perceptual cues that are salient enough to discriminate between two sounds may not be sufficient for stream segregation in electric hearing. Cooper and Roberts (2007) showed that pitch ranking scores of A and B tones were closely correlated with the percentage of time the CI users judged ABA triplets to be in two different streams. Based on this correlation, they argued that CI users were using pitch-discrimination to perform the stream segregation task, and were not truly performing stream segregation. Additionally, they did not see an effect of presentation rate on the ability to stream ABA triplets. Their pitch ranking results appear to lead to similar conclusions as our electrode discrimination results—that place pitch cues, though available, may not be effectively utilized for streaming. While not a direct measure of electrode discrimination, Hong and Turner (2006) compared normalized time delay thresholds in the IRD task to electrode separation between successive A and B tones to explore the potential role of place pitch cues. The electrodes stimulated by A and B tones were estimated based on the frequency allocation table. For some patients, electrode separation was significantly correlated with delay thresholds, suggesting that place pitch cues may have been sufficiently salient for streaming. Our study did not specifically examine pitch perception by CI listeners, but rather quantified their ability to tell loudness-balanced stimulation on two electrodes apart based on all available cues. We note that NH listeners have frequency difference limens (*f*0DLs) of approximately 1% (e.g., Qin and Oxenham, 2005); yet such small differences may not necessarily be sufficient to support stream segregation. NH listeners report perceptual segregation starts becoming evident around an eight semitone difference between noiseband stimuli (Bregman *et al.*, 2001), with greater separation in semitones leading to greater perceptual segregation for both tonal and noiseband stimuli (Dannenbring and Bregman, 1976; Bregman *et al.*, 2001). CI users can have difference limens an order of magnitude higher (e.g., Zeng 2002). Even larger perceptual differences, such as those introduced via the electrode discrimination task, should theoretically allow for stream segregation in CI users. Based on our results, this is not necessarily the case for all CI users.

The lack of a correlation between electrode discrimination and IRD thresholds in the CI users' data also suggests that gap discrimination between the A and B stimuli did not play an important role in the present study. As discussed in the Introduction, gap detection thresholds increase with increasing across-channel perceptual distance in CI listeners (Chatterjee *et al.*, 1998; Hanekom and Shannon, 1998). In addition, there was no correlation between the raw IRD threshold when A and B electrodes were the same (within channel discrimination) and the slope of the IRD vs B frequency functions for the CI data. This provides further support that the IRD task does indeed reflect streaming abilities rather than rhythm discrimination or gap detection.

In NH listeners, cues such as timbre readily contribute to stream segregation, as demonstrated in the noiseband experiments in the current study. The NH listeners' ability to perform stream segregation even when the temporal fine structure of the signal was considerably degraded and the spectral bandwidth broadened, suggests that the intact auditory system, even when aging, does not require very fine-grained information about the difference between two signals in order to form independent perceptual streams in a sequential streaming task. To the extent that the IRD task reflects sequential stream segregation, it appears that on average, CI listeners are in fact able to segregate perceptual streams despite degraded pitch cues, particularly when controlled stimuli are presented via a research interface. However, unlike the NH listeners' performance, considerable intersubject variability was observed in our CI participants, with some subjects completely unable to segregate the streams.

Another difference observed between the CI and NH listeners' performance was the asymmetry between apical and basal locations of the B stimulus and stream segregation in the CI population, with basal electrodes showing significantly greater segregation relative to the reference than apical electrodes. This asymmetry was not apparent in the electrode discrimination data in the same CI subjects, nor in the NH listeners' data with acoustic stimuli. Although no clear explanation for this observation is evident to us at present, the result further underscores the differences between electric and acoustic hearing, and reinforces the relative separation between electrode discrimination and stream segregation tasks.

One limitation of the present study is that we did not investigate the temporal “build-up” of stream segregation (Anstis and Saida, 1985; Cusack *et al.*, 2004; Roberts *et al.*, 2008). The experimental procedure was extremely time-consuming and participants were not available for additional tests. Other tests of stream segregation include the perceptual flip between an integrated-stream and segregated-stream state of perception that is experienced by listeners with long-duration sequences (Anstis and Saida, 1985; Cooper and Roberts, 2007). Such flips of perceptual state between two perceived images have been encountered in vision, e.g., Edgar Rubin's face/vase illusion where a visual ambiguity exists between perceiving two faces or a single vase. Although such subjective experiences are important hallmarks of auditory scene analysis, the scope of the present study was too limited to include them. However, in future studies we plan to include a smaller parameter set, which may allow the test of build-up of perceptual streams. Given the present results, the most parsimonious conclusion is that some CI listeners show evidence for partial stream segregation or a mechanism that is a precursor to streaming that is reflected in the IRD task, but the objective of this study was not to provide definitive evidence for the full set of phenomena that accompany stream segregation in NH listeners. Even if the IRD task reflects only elements of full stream segregation, CI listeners who show greater perceptual segregation are likely to be at an advantage in noisy backgrounds relative to those who show poorer segregation in this task (e.g., Hong and Turner, 2006). Thus, even though definitive conclusions regarding full stream segregation cannot be drawn from the present study, the results are likely to contribute to a deeper understanding of the true limitations of CI listeners in competing backgrounds.

In conclusion, our results indicate that age-related declines in temporal coding do not interfere with stream formation in NH listeners, and that electrode discrimination and gap detection cannot account for the results in CI listeners. Finally, we have found significant differences between patterns obtained with rigorously controlled stimuli presented through a research interface and stimuli presented through patients' speech processor.
